# Evaluation of point‐of‐care testing device for anemia detection: A cross‐sectional method comparison study from Thailand

**DOI:** 10.1002/jcla.23976

**Published:** 2021-08-24

**Authors:** Wittawat Chantkran, Pitipat Jamnarnwej, Pipat Sritanabutr, Pasra Arnutti

**Affiliations:** ^1^ Department of Pathology Phramongkutklao College of Medicine Bangkok Thailand; ^2^ Department of Clinical Pathology Phramongkutklao Hospital Bangkok Thailand

**Keywords:** bland‐Altman analysis, comparison study, interclass correlation coefficient, mission ultra Hb testing system, pearson correlation coefficient, point‐of‐care testing device, receiver operating characteristic curve, sensitivity, specificity, sysmex XN‐3000

## Abstract

**Background:**

A comparison study is crucial before launching a new medical device; therefore, we compared the Mission Ultra Hb Testing System with the Sysmex XN‐3000 automated hematology analyzer in Thai adult males and non‐pregnant adult females.

**Methods:**

Parallel studies were conducted using discarded venous K2‐ethylenediaminetetraacetic acid samples from participants requiring hematological investigations. According to the World Health Organization criteria, the participants were categorized as overall, anemia, and non‐anemia for analysis.

**Results:**

Three hundred participants were included in this study. In all participants, near‐perfect correlation and agreement were observed between the two methods for Hb measurement (*r* = 0.963, *p* < 0.001) with an interclass correlation coefficient (ICC) of 0.981 (95% confidence interval [CI]: 0.976–0.985) and Hct measurement (*r* = 0.941, *p* < 0.001) with an ICC of 0.965 (95% CI: 0.956–0.972). The sensitivity and specificity of the device in detecting anemia were 86.2% (95% CI: 79.7–91.2) and 98.6% (95% CI: 95.2–99.8), respectively. The area under the curve was 0.976 (95% CI: 0.963–0.989). The device showed average biases of 0.76 g/dl (95% limits of agreement [LOA]: −1.03 to 2.54) for Hb measurement and −2.73% (95% LOA: −9.28 to 3.82) for Hct measurement in all participants.

**Conclusion:**

Agreement between the Mission Ultra Hb Testing System and Sysmex XN‐3000 was observed. The device was excellent for detecting anemia. However, the essential evidence showing biases of the Hb and Hct measurements obtained from the device was revealed. Laboratory interpretation should be carefully performed, particularly at the near cut‐off values.

## Graphical abstract

The Mission Ultra Hb Testing System, as a tool for anemia detection, was compared with the Sysmex XN‐3000 automated hematology analyzer, as the reference, in Thai adult males and nonpregnant adult females. Near‐perfect correlation for hemoglobin (Hb) and hematocrit (Hct) measurements between the two methods was observed (Figure A). A high AUC value indicated that the accuracy of the device was outstanding in its overall performance for detecting anemia (Figure B).

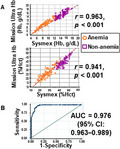

## 1 INTRODUCTION

Anemia is a serious public health problem worldwide. Based on data from the Global Burden of Diseases, Injuries, and Risk Factors Study, it affected approximately 32.9% of the world's population in 2010.^1^, ^2^ Interestingly, it has been reported that low‐ and middle‐income countries have the highest prevalence of anemia.^2^ In Thailand, the prevalence of anemia varies from 11.9% to 53.3% according to population and geographic distribution.^3^ Nevertheless, the reported prevalence may be underestimated due to poverty and the lack of opportunities for people residing in remote areas to access the public health system.

Several methods are commonly used to assess an individuals’ hemoglobin (Hb) and hematocrit (Hct) levels, including automated hematology analyzers, microhematocrit centrifuge, gravimetric copper sulfate method, cyanmethemoglobin method, and color code Hb estimation.^4^, ^5^, ^6^, ^7^ Although automated hematology analyzers are the gold standard and produce significantly accurate results for diagnosing anemia, their availability is limited to the regional, provincial, and district hospital levels in Thailand. The microhematocrit centrifuge must be of good quality and should be well maintained to provide accurate results. Moreover, it is not practical to transport the centrifuges to the remote areas of Thailand where an electrical power network supply may be inaccessible. The cyanmethemoglobin method is time‐consuming and labor‐intensive,^6^, ^7^ whereas both the gravimetric copper sulfate method and color code Hb estimation are subjective as estimator bias may be introduced.^4^, ^5^, ^7^ Therefore, in resource‐poor settings, a handheld or portable point‐of‐care testing (POCT) device for Hb and Hct level assessment potentially plays a key role as a tool for anemia detection, improving the problems of delayed diagnosis and interventions that lead to increased morbidity and mortality. Additionally, it could be a powerful tool used in demographic and health surveys for a nationwide epidemiological study.

However, before launching into implementation, a comparison study between a new POCT device and the reference method is crucial. In this study, we compared the Mission Ultra Hb Testing System, as a tool for anemia detection, with the Sysmex XN‐3000 automated hematology analyzer, as the reference, in adult males and non‐pregnant adult females, who constitute the majority of the Thai population. Information from the study could elucidate whether such a POCT device provides sufficiently accurate results for anemia detection.

## 2 MATERIALS AND METHODS

### 2.1 Study setting

This was a prospective and hospital‐based study conducted at the Internal Medicine Outpatient Clinic and the Department of Clinical Pathology, Phramongkutklao Hospital which is located in Bangkok, the capital city of Thailand. This study was reviewed and approved by the Institutional Review Board of the Royal Thai Army Medical Department (approval number S025b/64_Exp). This study was conducted in accordance with the principles of the Declaration of Helsinki. Informed consent was obtained from all participants included in the study.

### 2.2 Study participants and design

The study subjects were adult males and non‐pregnant adult females aged 18 years and older who required hematological investigations during their routine visits to the Internal Medicine Outpatient Clinic. The sample size was calculated using the prevalence of anemia in the Thai working‐age population of 17.3%^8^; estimated sensitivity and specificity of 98% and 95%, respectively^7^; the level of significance of 5%; and the level of estimation error of 5%. We estimated that at least 175 participants were needed, and at least 89 anemic patients were included. Venous K2‐ethylenediaminetetraacetic acid (EDTA) samples (Greiner Bio‐One, Chon Buri, Thailand) were sent to the Department of Clinical Pathology to be tested for clinical care using the Sysmex XN‐3000, then the discarded samples were immediately given to medical technologists for further testing, so that no extra blood was collected. Before participating in the study, the medical technologists were trained on the use of a Mission Ultra Hb meter based on the manufacturer's specifications and were blinded to the results from the Sysmex XN‐3000. Using 4 µl of discarded blood, parallel tests were performed using the Mission Ultra Hb meter within a maximum of 4 h of sample collection each day. For data analysis, the participants were categorized as overall, anemia, and non‐anemia. Anemia was defined according to the World Health Organization (WHO) criteria, that is, Hb level below 12.0 g/dl in non‐pregnant adult females and below 13.0 g/dl in adult males^9^ based on the results from the Sysmex XN‐3000, which was the reference method in this study.

### 2.3 Point‐of‐care testing method

The Mission Ultra Hb Testing System (ACON Laboratories, Inc., San Diego, CA, USA) consists of a handheld meter and disposable test strips. One lot of Mission Ultra Hb test strips was used (REF. C131‐6011, LOT. BHB0050001) throughout the study. The device uses the principle of electrochemistry for Hb detection,^10^ while Hct is measured by electrical impedance.^11^, ^12^ There are three modes available for blood sample testing, including whole blood anticoagulated with K2‐ or K3‐EDTA, or capillary blood.

### 2.4 Automated reference method

The Sysmex XN‐3000 hematology analyzer (Sysmex Corp., Kobe, Japan) is an automated blood cell counter for diagnostic use in clinical laboratories. Sysmex XN‐3000 uses cyanide‐free sodium lauryl sulfate (SLS) to determine Hb levels, whereas Hct level is measured based on the principle of hydrodynamic focusing.^13^

### 2.5 Quality control

The function of the Mission Ultra Hb meter was checked daily by measuring the three‐set standards of known concentration (low, normal, and high) provided by the manufacturer. Three‐set controls (low, normal, and high) were run daily to ensure adequate functionality of the Sysmex XN‐3000. The Sysmex XN‐3000 is under the Department of Clinical Pathology, Phramongkutklao Hospital, which receives International Organization for Standardization (ISO) 15189:2012 and ISO 15190:2003 certifications (valid until December 2022).

### 2.6 Statistical analyses

The data were analyzed using Stata Statistical Software Release 17 (StataCorp LLC, College Station, TX, USA). Scatter diagrams of the Pearson correlation and Bland‐Altman plots were visualized using MedCalc software version 20.0.5 (MedCalc Software Ltd., Ostend, Belgium). Demographic characteristics were analyzed using descriptive statistics. The outcomes are presented as numbers and percentages for categorical data and as means and standard deviations (SDs) for continuous data. Within‐group comparison (Mission Ultra Hb vs. Sysmex) was performed using the Wilcoxon signed‐rank test. The correlation (*r*) between the Hb or Hct values obtained from the Mission Ultra Hb meter and the Sysmex XN‐3000 was evaluated using the Pearson correlation coefficient. Agreement between the test methods was assessed using the interclass correlation coefficient (ICC) and the Bland‐Altman analysis.^14^ For the Bland‐Altman plot, the differences between each pair of measurements (Hb [Mission Ultra Hb–Sysmex] or Hct [Mission Ultra Hb–Sysmex]) were plotted on the vertical axis against the averages of the pair (Hb [Mission Ultra Hb + Sysmex]/2 or Hct [Mission Ultra Hb + Sysmex)/2]) on the horizontal axis, and the mean ± standard deviation (SD) of the differences and 95% confidence interval (95% CI) of the limits of agreement (LOA) (95% LOA) were calculated. The diagnostic accuracy of the device for detecting anemia was determined by sensitivity, specificity, predictive values, and the area under the receiver operating characteristic (ROC) curve (AUC). The level of statistical significance was set at *p* < 0.05.

## 3 RESULTS

### 3.1 Summary statistics of the participants and the hemoglobin and hematocrit levels

The summary statistics of the participants and the Hb and Hct levels are presented in Table 1. Anemia was defined according to the WHO criteria,^9^ based on the results from the Sysmex XN‐3000. A total of 300 participants were involved in the study and were aged between 20 and 94 years, with 50.7% of them being anemic. Males accounted for 60.7% of the participants. Unsurprisingly, anemia was more prevalent among females (63.6%) than among males and it was more commonly found in older participants. Considering each subpopulation, the results of the Hb level (g/dL) obtained from the Mission Ultra Hb meter were higher than those obtained from the Sysmex XN‐3000 in all subpopulation categories (*p* < 0.001). In contrast, compared with the Sysmex XN‐3000, the percentages of Hct obtained from the Mission Ultra Hb were lower in all subpopulation categories (*p* < 0.001).

**TABLE 1:** Summary statistics of the participants and the hemoglobin and hematocrit levels

	Overall n = 300	Anemic n = 152	Non‐anemic n = 148
Sex			
Male	182 (100%)	77 (42.3%)	105 (57.7%)
Female	118 (100%)	75 (63.6%)	43 (36.4%)
Age			
Mean ±SD	47.85 ± 24.7	58.66 ± 20.5	36.76 ± 23.76
Range	20–94	21–94	20–91
Hemoglobin level (g/dl)			
Mission Ultra Hb			
Mean ±SD	12.31 ± 3.25	9.74 ± 2.23	14.96 ± 1.55
Range	4.7–18.3	4.7–14.2	11.6–18.3
Sysmex XN−3000			
Mean ±SD	11.56 ± 3.39	8.66 ± 1.94	14.54 ± 1.34
Range	4.5–17.9	4.5–12.9	12–17.9
Hematocrit level (%)			
Mission Ultra Hb			
Mean ±SD	32.43 ± 8.41	26.2 ± 6.42	38.82 ± 4.5
Range	13.8–50.5	13.8–43.2	30.5–50.5
Sysmex XN−3000			
Mean ±SD	35.16 ± 9.67	27.14 ± 6.17	43.4 ± 4.05
Range	14.8–52.9	14.8–40.7	34.8–52.9

### 3.2 Correlation and agreement between the mission ultra Hb testing system and the sysmex XN‐3000

**FIGURE 1:**
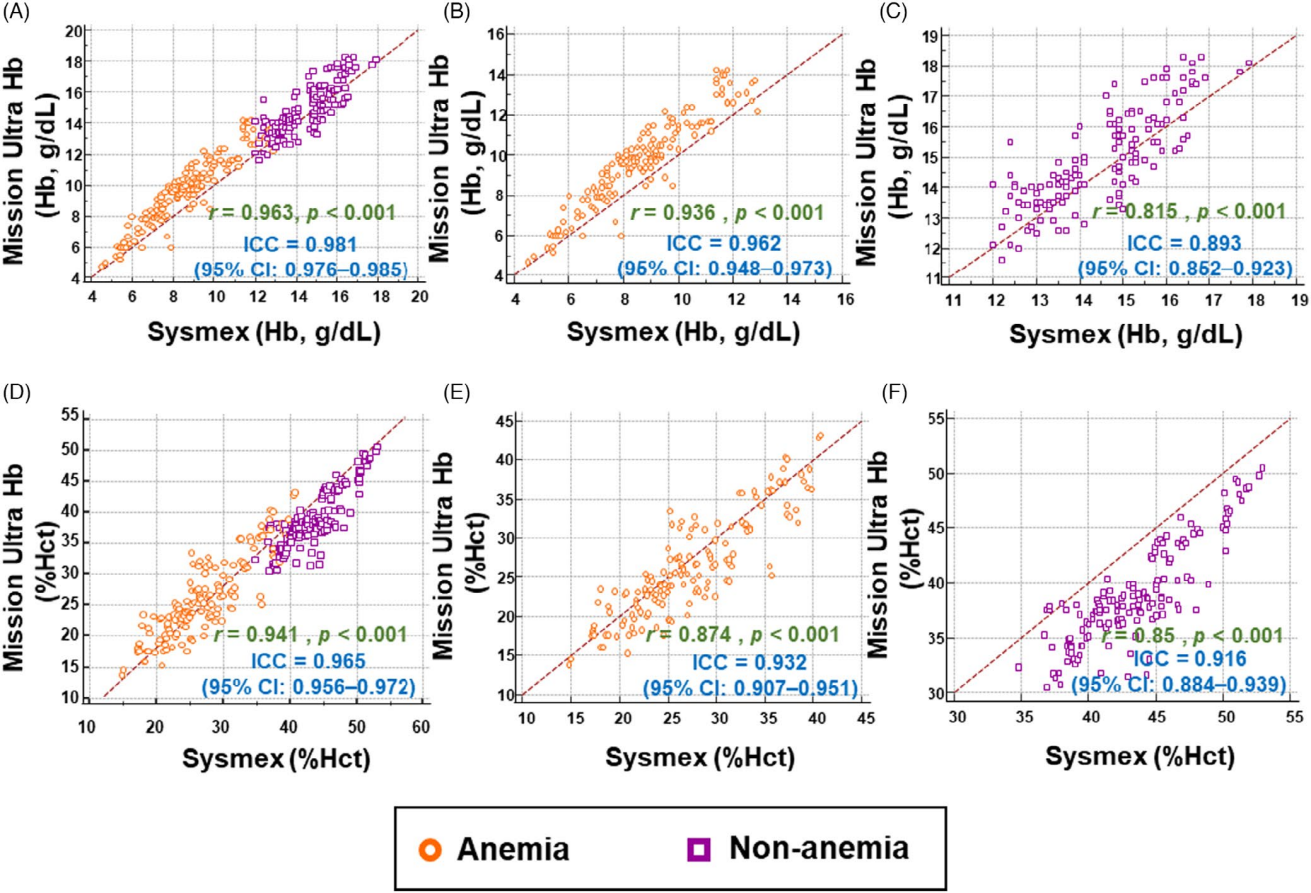
Correlation between the Mission Ultra Hb meter and the Sysmex XN‐3000. Scatterplots with the Pearson correlation coefficient (*r*) and the interclass correlation coefficient (ICC) of the hemoglobin (Hb) level obtained from the Mission Ultra Hb meter and the Sysmex XN‐3000 in (A) all participants, (B) anemic participants, and (C) non‐anemic participants. Scatterplots with the Pearson correlation coefficient (*r*) and the interclass correlation coefficient (ICC) of the hematocrit (Hct) level obtained from the Mission Ultra Hb meter and the Sysmex XN‐3000 in (D) all participants, (E) anemic participants, and (F) non‐anemic participants

As measured by the Mission Ultra Hb meter and the Sysmex XN‐3000, in all participants, near‐perfect correlation and agreement were observed for Hb measurement (Figure 1A) and Hct measurement (Figure 1D). In anemic participants, excellent correlation and agreement were also observed for Hb measurement (Figure 1B) and Hct measurement (Figure 1E). Nevertheless, the correlation and agreement slightly decreased in non‐anemic participants for Hb measurement (Figure 1C) and for Hct measurement (Figure 1F).

**FIGURE 2:**
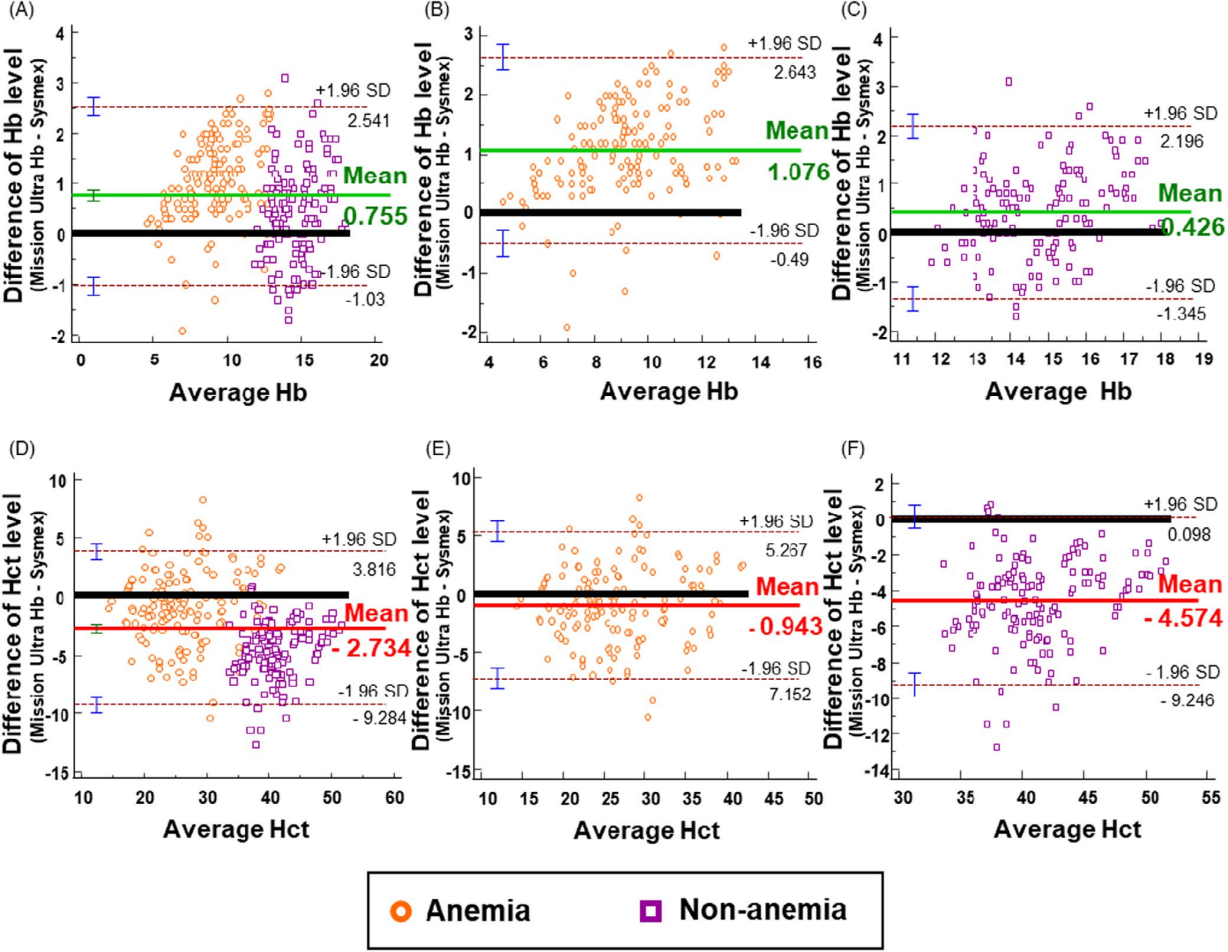
Agreement between the Mission Ultra Hb meter and the Sysmex XN‐3000. The Bland‐Altman plots of the differences of the hemoglobin (Hb) level obtained from the Mission Ultra Hb meter and the Sysmex XN‐3000 in (A) all, (B) anemic, and (C) non‐anemic participants. The Bland‐Altman plots of the differences of the hematocrit (Hct) level obtained from the Mission Ultra Hb meter and the Sysmex XN‐3000 in (D) all, (E) anemic, and (F) non‐anemic participants. The thick black lines depict a reference point (no difference). The green and red horizontal solid lines correspond to the average positive and negative biases, respectively. Two horizontal dashed lines represent the upper and lower prediction limits, corresponding to the 95% limits of agreement

Compared with the Sysmex XN‐3000, the Bland‐Altman plots revealed the essential evidence that biases of the Hb and Hct measurements obtained from the Mission Ultra Hb meter existed (Figure 2). Overall, positive biases for the Hb level (Figure 2A‐C) and negative biases for the Hct level (Figure 2D–F) were observed in all subpopulation categories. For Hb measurement, the average biases of the Mission Ultra Hb meter were approximately 0.76 g/dL (Figure 2A), 1.08 g/dL (Figure 2B), and 0.43 g/dL (Figure 2C), which corresponded to biases of 6.5%, 12.4%, and 2.9% of the average Hb level obtained from the Sysmex XN‐3000 in all, anemic, and non‐anemic participants, respectively. For Hct measurement, the average biases of the Mission Ultra Hb were approximately −2.73% (Figure 2D), −0.94% (Figure 2E), and −4.57% (Figure 2F), which corresponded to the biases of 7.8%, 3.5%, and 10.5% of the average Hct level obtained from the Sysmex XN‐3000 in all, anemic, and non‐anemic participants, respectively. According to the Clinical Laboratory Improvement Amendments (CLIA) 2019, proficiency testing for Hb and Hct measurements was defined as ±4% of the target.^15^ Therefore, the Mission Ultra Hb meter failed to meet the indicated cut‐off value for both Hb and Hct measurements.

### 3.3 Accuracy of the mission ultra Hb testing system as a tool for detection of anemia

**FIGURE 3:**
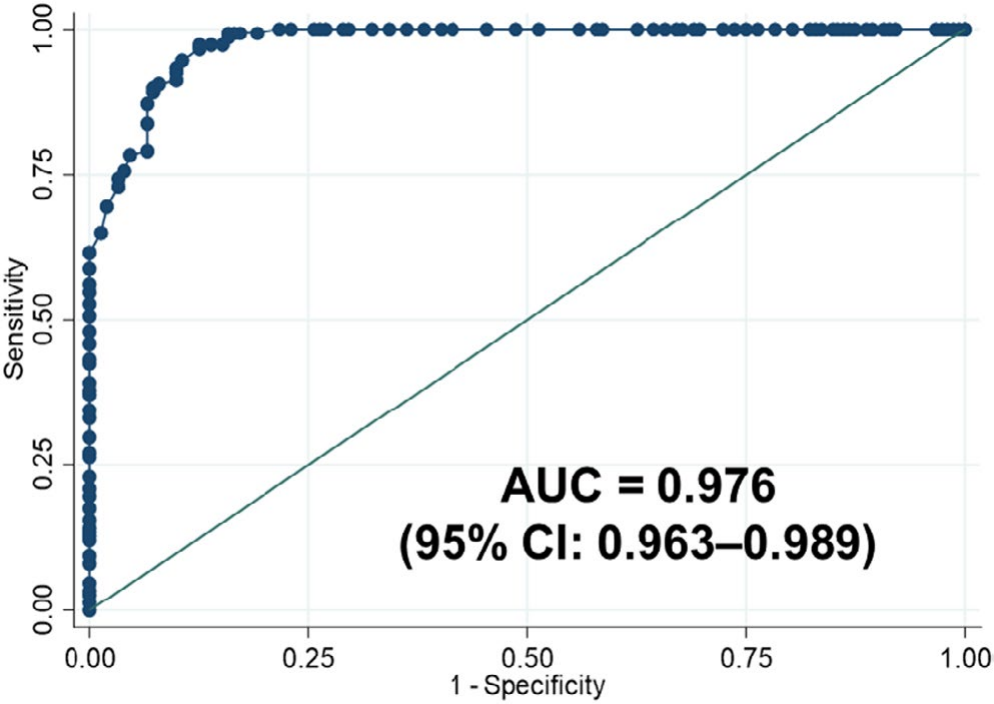
The receiver operating characteristic curve of the Mission Ultra Hb meter as a detective tool for anemia based on the hemoglobin level in all participants

Using the Sysmex XN‐3000 as the reference method and the WHO criteria^9^ for the Hb cut‐off values, the sensitivity and specificity of the Mission Ultra Hb meter in detecting anemia were 86.2% (95% CI: 79.7–91.2) and 98.6% (95% CI: 95.2–99.8), respectively. The positive predictive value was 98.5% (95% CI: 94.7–99.8), whereas the negative predictive value was 87.4% (95% CI: 81.4–92.0). In addition, the accuracy of the Mission Ultra Hb meter's ability to discriminate between the presence and absence of anemia was evaluated using the ROC curve. The ROC curve analysis showed a high AUC value (Figure 3).

## 4 DISCUSSION

Over the past 30 years, there has been a growing interest in the development of POCT for Hb and Hct level assessment. Apart from critical care settings in hospitals,^16^ handheld devices play an important role in field surveys for anemia screening.^17^ Accessibility to health care may be limited for some populations in developing countries, such as those living in rural areas and the border areas of Thailand. In resource‐poor settings, POCT largely functions as a key diagnostic tool. However, the crucial step is to verify the results prior to implementing the new device. In this study, we investigated the correlation and agreement between the Mission Ultra Hb Testing System and the reference method Sysmex XN‐3000 and assessed the accuracy of the Mission Ultra Hb meter in detecting anemia. To the best of our knowledge, this is the first correlational study of this handheld POCT device model.

A sample size of 300 participants in this study was sufficient to ensure the robustness of the results. Our medical technologists were well trained before participating in the study and strictly adhered to the manufacturer's recommendations. Quality control checks were performed regularly, further confirming the reliability of our results. The majority of the Thai population is working age^18^ with a continuously growing elderly population. Therefore, we focused on young, middle‐aged, and older adults in the present study.

A variety of statistical tests were used to determine the clinical efficiency of the device. Considering the Pearson correlation coefficient and the ICC, we observed that the correlation and agreement between the Mission Ultra Hb meter and the Sysmex XN‐3000 for Hb and Hct measurements were excellent in all participants (Figure 1A,D). The two statistical parameters consistently indicated that a correlation and agreement for both Hb and Hct measurements were slightly lower specifically in non‐anemic patients (Figure 1C,F) compared with other groups. In addition, the current study demonstrated that the Mission Ultra Hb meter has both high sensitivity and specificity in detecting anemia. Consistently, a high AUC value indicated that the accuracy of the device was outstanding in its overall performance for detecting anemia. The expense is significantly lower for the Mission Ultra Hb test compared with a complete blood count from an automated analyzer. Therefore, in terms of cost‐effectiveness, it is worthwhile to use a POCT device as an initial screening tool for anemia in resource‐poor settings.

Nevertheless, there was strong evidence indicating the presence of biases in the Mission Ultra Hb test. Using the Wilcoxon signed‐rank test, it was observed that the Hb level obtained from the Mission Ultra Hb meter was significantly higher compared with that obtained from the Sysmex XN‐3000. However, the Hct level obtained from the Mission Ultra Hb meter was significantly lower compared with that obtained from the Sysmex XN‐3000. This was reiterated by the Bland‐Altman plots, in which the biases were calculated and the Mission Ultra Hb meter failed to meet the ±4% limits according to CLIA 2019^15^ for both Hb and Hct measurements in almost all subpopulation categories. Regarding this issue, the near cut‐off values of Hb and Hct obtained from the Mission Ultra Hb meter must be carefully interpreted. In other words, there was a gray zone for undetermined results that anemia cannot be ruled out, that is, in general, approximately 2.6 g/dL (upper LOA, Figure 2B) above the Hb cut‐off values for anemia according to the WHO criteria.^9^ In contrast, in the case of serial Hct monitoring in critical care settings, a blood transfusion may be prescribed unnecessarily using a device with negative bias (Figure 2F). Another point of concern is pre‐analytical errors. For instance, we experienced inconsistent results when small air bubbles were introduced to a disposable test strip or when the mode of blood sample testing on the device was incorrectly selected. Hence, strict adherence to the manufacturer's recommendations is pivotal, and operators must pay careful attention while performing the test.

Our study had some limitations. First, pregnant females and children were not included; therefore, our results cannot be generalized to all subpopulations. Second, we used discarded venous K2‐EDTA samples; hence, the capillary mode of the device, which is mainly used in resource‐poor settings, was not evaluated. These issues need to be further examined in future studies.

In summary, venous Hb and Hct determinations using the Mission Ultra Hb Testing System were in acceptable agreement with the measurements obtained from the automated hematology analyzer. The performance of the device for detecting anemia was excellent. However, the essential evidence showing biases of the Hb and Hct measurements obtained from the device was revealed; therefore, laboratory interpretation should be carefully performed, particularly at the near cut‐off values. We recommend that further studies be conducted to compare this device with other POCT devices.

## CONFLICT OF INTEREST

The authors declare that no competing financial interests exist.

## AUTHOR CONTRIBUTIONS

W.C. developed the concept, collected and analyzed the data, and wrote and revised the manuscript; P.J. collected the samples and supervised the project; P.S. supervised the project; P.A. performed the experiment and supervised the project. All authors contributed and approved the final version.

## Data Availability

Data sharing not applicable to this article as no datasets were generated or analyzed during the current study.
